# *“Fala-M@no-COVID-19”*: technological development of a health navigation program for men during the pandemic

**DOI:** 10.1590/0034-7167-2022-0534

**Published:** 2023-12-04

**Authors:** Franciele Silva dos Santos, Lorena de Cerqueira Andrade Braga, Ramon Vinicius Peixoto da Silva Santos, Mary Karolyna Tavares Santos de Santana, Gildasio Souza Pereira, Vinícius de Olivera Muniz, Éric Santos Almeida, Anderson Reis de Sousa

**Affiliations:** I Universidade Federal da Bahia. Salvador, Bahia, Brazil; II Instituto Ensinar Brazil. Serra, Espírito Santos, Brazil

**Keywords:** COVID-19, Men’s Health, Health Education, Biomedical Technology, Nursing Process, COVID-19, Educación en Salud, Salud del Hombre, Tecnología Biomédica, Proceso de Enfermería, COVID-19, Saúde do Homem, Educação em Saúde, Tecnologia Biomédica, Processo de Enfermagem

## Abstract

**Objective:**

to develop a care-educational technology similar to a health navigation program for men during the COVID-19 pandemic.

**Methods:**

a methodological and qualitative study of a care-educational technology of health navigation program, structured by Program Development Cycle, with 16 patient navigators and 10 professional navigators. It used reflective thematic content analysis and an adaptation model for data processing.

**Results:**

the “Fala-M@ano-COVID-19”; navigation program was developed by: I) Observation of reality, problem mapping, needs assessment: content selection, creation of domains and questions; II) Theoretical-conceptual and methodological definition, creation of product under the elaboration of care plans, based on theory, process and taxonomies by a flowchart of operationalization of actions; and III) Self-assessment: qualitative research with professional navigators.

**Final considerations:**

the technology developed, with theoretical and methodological support, allowed to derive a viable navigation program compatible with reality based on the audience’s needs.

## RESUMO

**Objetivo:**

desenvolver uma tecnologia cuidativo-educacional do tipo programa de navegação em saúde para homens na pandemia de COVID-19.

**Métodos:**

estudo metodológico e qualitativo de uma tecnologia cuidativo-educacional do tipo programa de navegação em saúde, estruturado pelo *Program Development Cycle,* com 16 navegandos e 10 navegadores. Usou análise de conteúdo temática reflexiva e modelo de adaptação para tratamento dos dados.

**Resultados:**

o programa de navegação **“**Fala-M@ano-COVID-19” foi desenvolvido por: I) Observação da realidade, mapeamento do problema, levantamento das necessidades: seleção de conteúdo, criação de domínios e perguntas; II) Definição teórico-conceitual e metodológica, criação do produto sob elaboração de planos de cuidados, pautados em teoria, processo e taxonomias por um fluxograma de operacionalização das ações; e III) Autoavaliação: pesquisa qualitativa com os navegadores.

**Considerações finais:**

a tecnologia desenvolvida, com suporte teórico e metodológico, permitiu derivar um programa de navegação viável e compatível à realidade com base nas necessidades do público.

## RESUMEN

**Objetivo:**

desarrollar una tecnología cuidado-educativa similar a un programa de navegación en salud para hombres durante la pandemia de COVID-19.

**Métodos:**

estudio metodológico y cualitativo de una tecnología asistencial-educativa como un programa de navegación en salud, estructurado por el Ciclo de Desarrollo del Programa, con 16 navegandos y 10 navegadores. Se utilizó el análisis de contenido temático reflexivo y un modelo de adaptación para el procesamiento de datos.

**Resultados:**

el programa de navegación “Fala-M@ano-COVID-19”; fue desarrollado por: I) Observación de la realidad, mapeo de problemas, evaluación de necesidades: selección de contenidos, creación de dominios y preguntas; II) Definición teórico-conceptual y metodológica, creación del producto bajo la elaboración de planes de cuidados, fundamentados en teoría, proceso y taxonomías mediante un diagrama de flujo de operacionalización de acciones; y III) Autoevaluación: investigación cualitativa con navegadores.

**Consideraciones finales:**

la tecnología desarrollada, con sustento teórico y metodológico, permitió derivar un programa de navegación viable compatible con la realidad a partir de las necesidades del público.

## INTRODUCTION

Non-pharmacological measures to face the COVID-19 pandemic were essential in the context of this health emergency, given the magnitude and repercussions on health, whether at the individual, collective or environmental levels^(1)^. In view of this, the Pan American Health Organization (PAHO) released a guide for the application of these non-pharmacological measures aimed at vulnerable population groups in the context of COVID-19^(2)^. In this way, using educational resources, mediated by technologies, such as platforms in a virtual environment, has been widely used as a means of preventing and reducing the burden of illness due to COVID-19. Thus, it ensured transmission chain mapping, control and follow-up, with monitoring of cases and deaths, in addition to contributing to health promotion, with an increase in literacy levels, facing misinformation and in care production^(3)^.

In the meantime, there was evidence of a severe impairment of men’s health situation overview, with male subjects being more affected by COVID-19 in different parts of the world, with emphasis on the significant prevalence observed among men who resided in the Brazil. This has been related to a combination of biological (genetics, hormones), behavioral (eating habits, alcohol consumption, smoking and health care) and cultural (social construction of masculinities) factors that contribute to the fact that being a man is considered an amplifying factor of risk of illness^(4-6)^.

In order to face and transform this scenario, several initiatives were adopted by health authorities in different countries, most of them focused on disease treatment. In Brazil, on the other hand, government strategies aimed at health promotion, care production, communication and health education proved to be limited and fragile, which was reflected in the increase in male morbidity and mortality due to this type of cause^(7)^. Furthermore, scientific literature^(8)^ has indicated the presence of multiple disaggregating factors related to COVID-19 for health in its different dimensions (physical, sexual, affective-marital, family, work, psycho-emotional/mental, spiritual and bioenergetic), which justifies the need to develop care-educational interventions and technologies that provide means of mitigating and coping with the impacts of the pandemic on men’s lives and health.

In this context, the creation of different support and care strategies in virtual environments stands out, to the detriment of routine on-site care and/or traditional care/monitoring model^(9)^, offered through health navigation programs, advice and/or remote monitoring, telenursing, teleconsultation and information systems. All have the objective of systematizing, keeping sensitive data and ensuring intercommunication between the various actors in the health system^(10)^.

There is evidence^(11)^ that health programs focused on supporting technology-mediated care contribute to effective coping, helping communication and care education in health and emotional regulation. As for the structural aspects of viability, feasibility and cost-effectiveness, virtual health programs allow the performance of multiple tasks in real time, rationalization of expenses destined to health resources, sustainability and sufficiency, and have proven to be contributory in the COVID-19 pandemic, given the subsidy given to the “care-education” process of male individuals^(11-12)^. Despite this, there are still gaps in the literature regarding actions aimed at the male population, in the context of the COVID-19 pandemic, which have as their centrality support for health care, which justifies this study and reveals its relevance and social utility.

## OBJECTIVE

To develop a care-educational technology (CET) of a health navigation program for men in the COVID-19 pandemic.

## METHODS

### Ethical aspects

The project was approved by the Research Ethics Committee. Because it involves human beings, the guidelines of Resolutions 466/2012 and 674/2022 and Circular Letter 2/2021, belonging to the Brazilian National Health Council, in addition to the General Data Protection Law, were strictly complied with. Participants’ consent was obtained by accessing, reading and signing the Informed Consent Form (virtual-picture version). Guidance was provided regarding ethical care, surveillance and protection of data derived from research in virtual environments. Participants’ anonymity was preserved by identifying codenames such as Patient navigator_01 (NAV_01), Patient navigator_02 (NAV_02) and so on. The team of professional navigators was named PROFESSIONAL NAVIGATOR_01, PROFESSIONAL NAVIGATOR_02 and so on.

### Theoretical-methodological framework

Technological creation was structured in the theoretical-conceptual perspective of CET, which can be understood as a process that contributes to the development of nursing as a science and for advances in the profession, with a viable application, low operational cost and as a cyclical process between care-education-care, involving who is cared for (individual) and who educates for care (health professionals). Furthermore, CET makes use of the understanding of praxis, a term used to situate practice - an activity carried out in a conscious and oriented manner that involves objective and subjective dimensions^(13)^.

CET was operationalized through a remote navigation program in health, with the elaboration of a protocol containing the objectives, activities and actions to be carried out as well as the description of the respective stages, those involved and their attributions for program development, which had throughout its CET intervention process inspired by the proposal of navigation in health^(14)^. Thus, Program Development Cycle^(15)^ principles are observed, which were adapted to the context of the program in question.

Adopting the model involved a process of reading the standardized language taxonomies of international documents (development manuals) and national scientific texts that addressed the program use, its limits, potentialities and the particularities of its application in the Brazilian reality. Moreover, constructs related to navigation in health arranged on the Navigation Needs Assessment Scale (EANN)^(14)^ were accommodated as a way of customizing actions to the chosen target audience.

### Study design

This is a national methodological study, with a qualitative approach, based on the development and implementation of a CET of a health navigation program, whose stages were redesigned with reference to the stages proposed by Polit and Beck for the construction of methodological studies^(16)^ and the Program Development Cycle^(15)^. Methodological research is circumscribed as an investigation that rigorously guides the constructive, organizational, development and validity stages of a research itself as well as its instruments and methods, providing solid, reliable and innovative results for the target audience. Moreover, it provides a lot of representation in nurses’ contemporary performance in producing care that contributes to the population’s health. Regarding the scenario and technology, it was developed via email.

### Setting and period

The study derives from research-matrix and extension project entitled “*Vivências de homens em contexto de pandemia do novo Coronavírus - Sars-Cov-2 (COVID-19) no Brasil: um enfoque para a saúde*”, linked to the *Universidade Federal da Bahia*, in operation since 2021. The team consisted of an undergraduate and a graduate student in nursing, two graduate students in physiotherapy, a graduate student in nutrition, a graduate student in medicine and four nurses: three masters’ and one PhD in nursing and health. Extension project activities, in particular, started in August 2021, extending until August 2022.

### Data source

The participants involved in the study comprised two distinct groups: the first was composed of sixteen adult Brazilian men (patient navigator); and the second was composed of six students and four nurses (professional navigators) residing in the state of Bahia. All experienced the proposed technology. To be a professional navigator, two inclusion criteria were involved: being students in health or nursing courses; and have been approved in the project selection process. As for patient navigators, the criteria were to be an adult male, residing in Brazil, with access to information and communication technology resources, to have an email address and to have participated in the main research project. Exclusion criteria were non-regularity in the research group meetings (professional navigator group) and lack of response to the invitation email (patient navigator group).

### Data collection and organization

**Figure 1:**
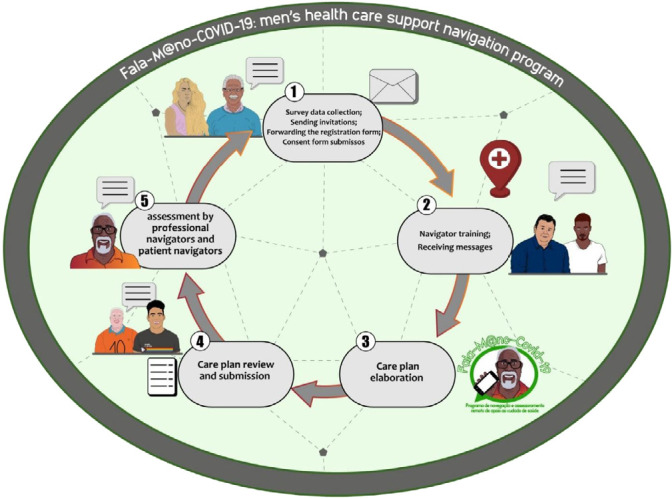
Explanatory flowchart of the development stages of a health navigation program’s praxis guided by the Program Development Cycle. Salvador, Bahia, Brazil, 2022

The professional navigator group formation was based on the public selection of students and invitation to professionals. To form the patient navigator group, several awareness raising strategies were used with a restricted approach and exclusively via email. For this, specific cards were prepared and disseminated on social networks such as Instagram^®^ (@cuidadoasaudedehomens), in specific groups on Facebook^®^, Telegram^®^ and WhatsApp^®^, and through registered emails, in order to boost program dissemination. The cards contained the program information, as shown in Figure 1. It should be noted that the collected emails were registered spontaneously, giving consent to be contacted again. Overall, 500 emails were sent, obtaining seven favorable responses and one withdrawal. Thus, the professional navigator team’s work was carried out based on the distribution into three subgroups, who were responsible for the “Fala-M@ano-COVID-19”; program’s operational activities. Three tests were carried out internally by the team to validate the protocol, followed by weekly meetings to check and resolve pending issues.

The domains that constituted the navigation program were defined from standardized questions, forged within the scope of the set of nursing language classification systems (NANDA-I taxonomies, Nursing Diagnoses, Nursing Interventions Classification (NIC), Nursing Outcomes Classification (NOC) Classification System and Adaptation Theory), in addition to scientific evidence, guidance from technical manuals from health authorities, clinical guidelines and other guidelines. These proposed interventions were based on measurable, validated and effective results, which contributes to the reduction of biases, providing better support for health care.

In this way, the respective care plans were elaborated, but assuming customization, according to the demands and needs expressed by patient navigators, with the help of a previous sociodemographic characterization. Plan documentation was done in a specific model for recording the demands, guidelines and expected results, kept in the drive with restricted access, constituting a digital medical record. Monitoring worksheets to control sending and receiving emails were also adopted to manage interactions and interventions.

Thus, the program was developed, comprising the following moments: 1) Survey of needs, through questions that were prepared in line with NANDA-I domains, sent via email to navigators, thus allowing that, from interaction, a diagnosis of their demands; 2) Assessment of patient navigator responses and interactions by the professional navigator team, with identification and discussion of respective explicit needs, followed by content analysis according to nursing knowledge; 3) Elaboration of an individual care plan by the professional navigator team, with guidelines, suggestions and recommendations relevant to assessed needs; and 4) Feedback referral, in the form of individualized care plans, with subsequent verification with patient navigators and assessment of the set of plans by the professional navigator team.

### Data analysis

Reflective thematic content analysis^(17)^ was used to interpret patient navigators’ speeches and a theoretical framework, according to the four modes of Roy’s adaptation model: physiological and physical; group self-concept identity; role function; and interdependence^(18)^. Content analysis was also used in navigators’ self-assessment.

## RESULTS

Overall, there were 16 participants. The categories corresponding to the stages of development of “Fala-M@ano-COVID-19”; will be presented below.

### 1. Observation/analysis of reality, mapping the problem and surveying the target audience’s needs

Within stage 1, technology development involved data thematic analysis from participants, which gave rise to a unique thematic category that explained the experience of men in the pandemic in relation to health care, which revealed compliance with care practices mediated by virtual resources:

#### Experience of men in the pandemic and adaptive health care: pointing out possibilities for virtual support

[...] *I started to start an online psychological treatment.* (NAV_01)

[...] *I’ve been trying to take care of my mental health, participating in groups of men through social networks.* (NAV_02)

[...] *I feel that I have improved health care, as I started looking for more information on the internet, on reliable sites, leading me to prevent myself from COVID-19.* (NAV_03)

[...] *this initiative to think about men’s health is very important, because we are led to believe that we are strong and invulnerable, that we will handle everything. It would be great to be able to have more actions for men on social media.* (NAV_04)

Participants self-reported as homosexual (6; 100%), cisgender (5; 83.3%) and non-binary (1; 16.6%), aged 30-39 years (3; 50%), 40-49 years (2; 33.3%) and 50-59 years (1; 16.6%). They have different birthplaces, such as Camaçari/BA, Feira de Santana/BA, Salvador/BA, Goiânia/GO, Belo Horizonte/MG and Cabo Frio/RJ, in addition to being white (4; 66.6%) and brown (2; 33.3%). They are single (5; 83.3%) or married/stable union (1; 16.6%). None had children (6;100%), and all had completed secondary education (6;100%). They were employees of the federal, state or municipal government (4; 66.6%), in addition to liberal professionals (1;16.6%) and informal workers (1; 16.6%), with monthly income between 1-3 (2; 33.3%), 6-9 (2; 33.3%) and 9-12 (1; 33.3%) minimum wages. They worked 40 hours a week (2; 33.3%), with no fixed hours (2; 33.3%), in addition to 21-30 hours (1; 16.6%) and 31-40 hours (1 ;16.6). They owned their own house (3; 50%) or rented (3; 50%), in urban (5; 83.3%) and rural areas (1; 16.6%).

The observation of reality made it possible to derive the focus of attention of the “Fala-M@no-COVID-19”; program to be worked with the target audience, organized into ten thematic domains, with their respective objectives and standardized questions, responsible for mobilizing the interaction between professional navigators and patient navigators. Furthermore, the actual structuring of the developed CET was based on the theoretical-conceptual and methodological definition of the terms used and techniques employed. Thus, it was possible to build a survey of demands presented by the target audience, participant characterization and operational flowchart of health navigation actions and individual health support care plan (technological product).

### 2. Theoretical-conceptual and methodological definition - creation of a technological product: men’s health navigation program

The methodological development stage enabled the creation of technology based on a first mapping of the investigated phenomenon. For this, custom stages based on the originals are described below, and provided the functional structuring of the design of the navigation program created.

#### Operational flowchart of health navigation actions

Operationalization actions were carried out by the participation of working groups: Group A: sending an initial reception message from patient navigators via email; checking email and spam inbox to check responses; gathering responses and placing them in electronic records for each patient navigator and forwarding the responses to create care plan (return); Group B: organization of patient navigator responses in folders and control sheets; online drive file management; and care plan preparation (research of scientific evidence on the subject, investigation of diagnoses, results and nursing interventions, in order to meet the demands presented by patient navigators); Group C: care plan elaboration supervision; care plan internal validity (consensus meeting and content parameterization, request for external validity by specialized professionals, when necessary, final reading for alignment and customization (grammatical review, agreement on the use of terms/concepts, acronyms and references used)); and health navigation program management (quality, feasibility and sustainability assessment - permanence and/or withdrawal of patient navigators, motivation and/or demotivation of professional navigator team, awareness).

For action viability, weekly remote meetings were held with the purpose of building and refining the activities. The entire construction was taken to appreciation and approval by the professional navigator team, who made use of transfer of information and solving of doubts. In Figure 2, the explanatory flowchart of the stages of development and operationalization of health navigation program actions’ praxis is presented.

The program implementation proposed, as one of its products, the construction of individual care plans to support health, elaborated based on each domain and presented in a specific structure in a dialogic nature, containing guidelines directed to the demands identified in interaction with patient navigators, subsequently forwarded to each participant, individually, making a total of ten care plans forwarded per participant. Care plan design was aimed at achieving nurses’ awareness during their professional practice, with a view to promoting health and social support for men affected by the COVID-19 pandemic. Below, in Chart 1, a care plan model developed.

**Chart 1:** Individual health support care plan. Salvador, Bahia, Brazil, 2022

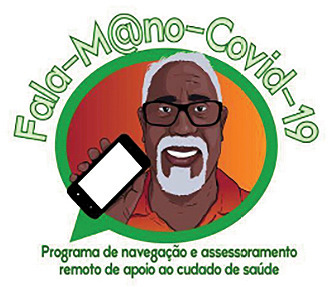	
**INDIVIDUAL HEALTH SUPPORT CARE PLAN**	
**DOMAIN 03: STRESS COPING/TOLERANCE**	
**Patient navigator 03:**	**Intervention date:** 07/26/2022. **Record:** via email.
**Demand/problem situation:**	**Message received after intervention 03 (patient navigator’s speech):** **Question 1:** […] *quite. I was already in a period of increased stress and anxiety, and the changes needed to accommodate the reality of the pandemic took away several of the things that helped me deal with this overload, mainly the separation of a living and working environment, limits on schedules and the free possibility to choose where to go.* **Question 2:** […] *quite. I am already naturally stress-prone, and with the addition of all the stressors that came with the pandemic and not being able to do a lot of the things I used to deal with stress made the situation worse, the moments of decompression decreased.* **Question 3:** […] *I have been forcing myself to have a definite and regular sleep schedule. Decreases the use of stimulants (caffeine, taurine…), reduces the number of hours of work per day, invests a lot in hobbies that can be done from home.* **Question 4:** […] *in general, so much for the expectation that, as a man, I have to fend for myself and be able to get by without asking for much help.* **Question 5:** […] *I feel that I have been greatly affected, mainly by the flood of information, and being constantly aware of certain things. Not only too much news (mostly bad) about COVID, but having to remember the subject for all activities and needing to police myself led to a lot of stress and anxiety when observing others’ attitudes and behaviors. I started to get extremely irritated and angry at seeing people not respecting protocols and using proven ineffective methods of prevention, to the point of losing my good mood for the day if I encountered a case of this type*.
**Support guidelines:**	**1. Do you think the context of the pandemic interfered with your mental health? if so, how?** **Guidance 1.1:** we are very sorry that this happened to you, and we would like to say that most of us had to face the new reality of working and living in the same space, controlling, as much as possible, our energy expenditure and the domestic environment. We hope that you have already managed to establish boundaries between the two worlds of “personal life” and that of “professional life”; if not, we advise you to try to create a routine, in an extraordinary life agenda, which can be created by reading the book “The power of action”, as it is a reading that encourages the performance of a series of exercises that will help you manage your time, organizing it for each activity of interest. We also suggest that you create different environments in your own home and avoid sleeping in the same room you use for work; also, practice meditation and guided imagery, join support groups, adopt a pet, perform psychotherapy with specialist professionals who work with cognitive-behavioral therapy, receive visitors, cook, move furniture a little and practice physical activities guided by video classes of exercises using your own body weight and dancing, which can help you control stress, anxiety and overload as well as reduce the triggers that lead you to these undesirable feelings. Having a corner to practice relaxation and self-knowledge activities with aromas to your liking, through incense or aromatic diffusers, is one more option that we can suggest as a technique to calm and reduce anxiety.
	**2. Have you been feeling affected by stress in this pandemic moment? If yes, tell me more about it.** **Guidance 2.1:** dealing with situations of stress overload ends up making us more susceptible to situations of anger, and it is up to us to know how to manage it and reduce the anxiety itself. We know that stressing out is not easy, but with effort, it becomes possible. We will try the best to help you! We advise you to try to create these moments of “decompression” in your daily life, reserving at least 10 minutes to listen to music of your choice that calms you down and that you enjoy, in addition to some reading (magazines, newspapers or books) that interests you and/or watching that series or movie that makes you feel good. Taking time for yourself is essential, don’t forget that. Additionally, we suggest you look for emotional support; practice actions that promote resilience, such as sharing the moments when you need to make important decisions in your life with loved ones (friends, family or neighbors with whom you have closeness and a certain intimacy), including asking for advice from older people who have more experience with life, increasingly strengthening your support system; take care of the environment in which you sleep so that it can be a pleasant place to have restful and regular sleep; avoid the abuse of alcohol; do not use illicit drugs; try to maintain a healthy diet (avoid processed and industrialized foods, give preference to legumes, fruits, vegetables, fibers and a controlled consumption of meat, giving preference to lean and white meat); and, finally, go for walks with light runs in open, airy places and close to nature.
	**3. What strategies have you used to deal with stress?** **Guidance 3.1:** very good, congratulations! These actions that you report make us very happy, because we understand that you are willing to improve your self-care, and this will generate good results for a self-modification of habits that can harm you, such as reducing the consumption of stimulants can improve your sleep. It’s good to know that you already carry out activities to face some perceived lack of feeling of comfort, relief or even transcendence in the physical, psycho-spiritual, environmental, cultural and social dimensions. Therefore, through activities for yourself, you will help yourself to achieve goals that will lead to improved and strengthened health. We advise you to continue like this, reducing stimulating drinks, resting and having fun. We would add (because it was not commented) that you invite friends to your house to have fun and talk with them, because good conversations are positive in our lives. We suggest, in addition to these activities and along with the actions you have already developed, that you attribute yourself to your own merit; control your energy expenditures; try to maintain a constant mood; take relaxing baths; facilitate for self-responsibility to be effective; and practice activities that raise your self-esteem and work on your spirituality, regardless of your belief or religion, and, of course, it’s always good to remember to do things that make you happy.
**Support guidelines:**	**4. Do you realize if being a man puts you in a problematic situation that affects your mental health? If yes, tell me more about it.** **Guidance 4.1:** unfortunately, this is still very present. We advise you to try not to pay too much attention to these adverse opinions. If you feel the need for something, seek help, without worrying about what others think. We understand that we men have carried this burden since the dawn of civilization, that men need to be the sole providers of the home and that, otherwise, society’s judgments begin to affect us, but we need to support each other to positively face these negative perceptions about our own worth in response to a current situation, also understanding that we do not need to be in control of every situation in our lives and that this cannot leave us feeling powerless. On the contrary, we need to build a self-awareness that we have predetermined roles for ourselves and that all our actions can generate a result in a meaningful way. In this way, you will be able to generate answers capable of putting into practice your chosen ethical and moral decisions and actions. For this, we suggest that you determine, as much as you can, what your true role is as a person in the environment you live in (clarify to yourself what your values are); value socializing with friends and family; build concrete and reciprocal relationships; practice art therapy and self-responsibility, as it will help you maintain balanced self-esteem; be self-effective when practicing activities of assertiveness and promoting hope for a better future; create alternatives that will help you, in case you need to face situations of grief (moments of sadness, hurt and maybe you feel sorry for something or someone); tell the truth and practice forgiveness when necessary; forgive; also practice memory therapy guided by a specialized professional.
	**5. During the pandemic, very common situations, such as social isolation, excessive information about COVID, loss of close people and others that you may have experienced, have had an impact on men’s mental health. Do you feel affected by any of these situations? Tell us more about it.** **Guidance 5.1:** we know that these moments make us very irritated and angry, because we can’t understand very well if people behave that way or if they really don’t have access to true information. However, we can help you to develop skills to deal with these stressors and choose, appropriately, your responses and behavior in the face of these situations using the available resources. Therefore, we are here to advise you, encourage you to recognize, express and relieve your feelings of anxiety, anger or sadness in an adaptive and non-violent way, having already a positive point, which is the fact that you know the source of your anger, and that’s great! We suggest that you do not give too much importance and limit yourself to having access to these frustrating situations. Here, we suggest that you practice activities that will help you to adjust to intense emotions, such as distracting yourself, appreciating and expressing what is funny and amusing, in order to establish relationships, relieve tension and release anger. Also, perform deep breathing exercises and keep a journal so that it is possible to control your ideas at that moment of peak anger and modify your behavior. Lastly, perform progressive muscle relaxation exercises.
**Expected results:**	It is hoped that this plan will encourage you to cope better in situations that cause you stress, anxiety and anger. We also hope to help you better understand your role as a human being and that you can count on people who have mutual affection and esteem for you and you for them.
^*^This care plan was developed by undergraduate nursing and physical therapy students, supervised by four nurses with master’s and doctoral degrees. A social worker and a psychologist were consulted. Project approved by the Research Ethics Committee, under Opinion CAAE 32889420.9.0000.5531 and 4.087.611. School of Nursing - *Universidade Federal da Bahia* (UFBA). R. Basílio da Gama, 241 - Canela, Salvador - BA, 40231-300.	

### 3. Self-assessment

The professional navigators’ content expressed the program’s self-assessment, expressing the sense of challenge in the face of the novelty of COVID-19, the involvement in navigation actions seen as aggregators and the limiting factors for the program’s operation, presented in the following narratives:

[…] *the challenge is relevant to the new, from conception to implementation, barriers emerged that were solved as a group. In addition, being involved in the search for reliable national and international evidence that would support the development of a program and meet the expectations of patient navigators, with minimal resources, was a very important experience.* (PROFESSIONAL NAVIGATOR_01)

[…] *we keep sending new reminders until we receive our first participants in the pilot project. I realize the magnitude of this project, which, due to the ten domains being worked on in a safe and responsible manner, will play a relevant role in reducing the problems experienced by Brazilians during and after the pandemic.* (PROFESSIONAL NAVIGATOR_2)

[…] *the technology that would carry out real-time interaction and approximation among participants of the process of the stage that we advanced had a great difficulty in pilot project implementation, which was the gathering of participants, as contacts were made via email and we defined monitoring for 48 hours, and the response was not as expected, due to the number of invitations sent.* (PROFESSIONAL NAVIGATOR_3)

## DISCUSSION

This study expressed as a central finding the essential elements for developing a health navigation program, with the scope of actions to support the care of men in the context of the COVID-19 pandemic. Such technological development was primarily based on the recognition of the health care scenario exercised by men in the pandemic. In this regard, all resources used seek to meet the specificities and their demands.

Patient navigation programs are associated with positive results in terms of expanding access to care, with reduced waiting time for diagnosis and treatment, in addition to favoring better adherence to triage, improving coordination and continuity of care. Despite this, there is still little evidence of its results outside the United States of America (USA)^(17)^. However, navigation programs aimed at other contexts, such as health promotion, encouraging self-care, changes in life habits, raising levels of health literacy and, even more, with a focus on gender, as in the case of men, are still scarce and deserve to be explored and expanded. Furthermore, there are different contributions of a health navigation program for patients or professional navigators. It is noteworthy that, for people with cancer, navigation is seen as valuable, with advances in the emotional support received, accommodation of information, assistance demands and problem solving and help in the logistics of treatments experienced^(14,18)^, as in the case of people living with diabetes^(19)^: improvement in the understanding of the disease; increased motivation for managing health care, which can be exploited in contexts of health crises, such as the COVID-19 pandemic^(20)^, Mpox, or humanitarian situations, such as the war in Ukraine.

Despite the COVID-19 pandemic and its persistence in some countries as Brazil, using non-pharmacological resources for coping is essential for achieving “post-pandemic resilience”, which has drawn the World Health Organization’s attention. In this regard, PAHO has reinforced the good use of Information and Communication Technologies, the creation of posters, videos and electronic message boards to promote improved access to safe and quality knowledge as well as contribute to the processes of detection, analysis, monitoring and follow-up of the population affected by the pandemic context^(2)^. From this perspective, CET has in its concept the involvement of a set of knowledge and scientific knowledge that are interrelated and are part of nursing professionals’ daily life who, consequently, outline the principles of human practice, accessed in the exercise of the profession^(13)^ and, therefore, with coherence and affinity with the scenario of a health crisis.

Using digital technologies has had a strong impact on health work, as it is increasingly being incorporated into these practices. The need for digital literacy on the part of nurses and other health professionals has been of relevance^(21)^. By encouraging self-care and changing habits, digital health care technologies are able to enhance subjects’ skills, controlling non-communicable diseases and helping in education, prevention and health promotion^(22-23)^, in addition to being essential for care production and health promotion for men. Thus, the knowledge and knowledge that make up CET go towards the process of caring/educating and educating/caring, in an intimate relationship between the subject and the other, in a given praxis that must be used consciously and guided by professionals^(13)^, which can be useful in contexts of changes in everyday life, given the emergence of new behaviors adopted by a community.

Governments around the world have been looking for solutions to maintain the public health system and its potential risks as a result of restrictions caused by the lockdown^(20)^. Accordingly, some of the solutions adopted are health technologies. Certainly, using health technologies during the COVID-19 pandemic period has positive effects on men’s health, highlighting their potential in care communication processes, emotional regulation and strengthening of support networks^(8)^. Furthermore, the inclusion of health technologies in pandemics makes it possible to care for individuals with high levels of anxiety or symptoms suggestive of COVID-19, which can be monitored remotely^(24)^.

When rethinking the impacts of such care technologies in the post-pandemic, it is assumed that they are important in the coordination of the health system’s different agents, since they have influence to reduce unnecessary referrals, reduce people’s physical circulation and guarantee equity and universality of access to health services^(12,25)^. Thus, CET can contribute to producing knowledge in nursing and guide professional practice, since it intertwines different theoretical and philosophical references based on the relationship between technologies and praxis^(13)^. In view of this, attention is called to consider the specificities of each group/target audience of the interventions. In the case of health navigation for men, it is necessary to analyze living conditions, digital literacy, socioeconomic vulnerabilities, avoiding exclusions.

The adaptation of technologies to assist in coping with COVID-19 cases has become fundamental for care practice^(26)^. Allied to innovation, it presents itself as an essential tool, since it is increasingly present in society, drawing a direct connection with the current pandemic scenario^(27)^. Likewise, in the West, the strategies of countries such as Italy, France, Latvia, Switzerland and the United Kingdom are successful in the good use of their own applications, a study reported^(28)^, and in China, smart technologies were used to support health actions during the pandemic^(29)^, which exposes a promising scenario for nursing and health^(30)^.

It is important to highlight several initiatives related to teleconsultation/telenursing as well as other ways, such as that of the Ministry of Health itself, which provided a telephone number (136), an online chat and a communication channel via WhatsApp^®^ precisely with the aim of facilitating communication between the patient and the health service, minimizing the risks of exposure and contagion^(31)^. In addition to this, in Brazil, the Federal Council of Nursing recognized digital health as a new specialty^(32)^. Thus, through these advances in technological and digital resource use by nursing professionals, it is believed that the conceptual approach of CET may be useful in nurses’ practice in the context of education and care (caring-educating and educating-caring), in the search for autonomy promotion, for the population’s empowerment and well-being in a given scope of the health-disease process, as in the case of men assisted by nursing professionals and other health areas, being the focus of the development of technologies-processes and products^(13)^.

In the disciplinary scope of nursing, exploring one’s own knowledge such as that arising from nursing theories, the Nursing Process and its taxonomies will contribute to strengthening the profession in technical and scientific terms^(3)^. In times of a pandemic, human responses may present many problems, which will become the object of nursing practice, such as the adaptive modes to be exercised by individuals, when confronted with expressive and complex stressful events, such as those caused in a pandemic, which will require support and professional attention to be provided to health professionals and vice versa, considering beings who care and who also need to be cared for^(33)^. Thus, it is recommended that non-pharmacological technologies be developed based on theoretical and physiological assumptions specific to nursing. Thus, creating well-integrated programs such as those already mentioned above, such as “Fala-Man@-COVID-19”;, could be useful in social support to populations and in teaching for self-management of health care. To this end, emphasis is placed on the need for intersectoral articulation, appreciation of teaching, service and community integration and professional and collaborative work in health technology development, as reported in this study.

### Study limitations

Technological development did not have funding, which impacted using other tools such as employment or creation of platforms, software and applications. Using emails as a means of establishing communication and conversation with participants resulted in delay in responses, lack of responses and operational failures, such as spam.

### Contributions to nursing

The study can have a satisfactory impact on the review of practices traditionally adopted by professionals and services, and on the improvement of health care networks and lines, through the implementation of complementary remote programs. It meets, in a biopsychosocial-spiritual way, the population’s adaptive modes and needs, acting with a focus on the expanded concept of health, by strengthening interdisciplinarity, interprofessionality, building skills in prescribing non-pharmacological care. Moreover, it expands the scope of care production in nursing and health, in view of male demands and advances in digital health in nursing.

## FINAL CONSIDERATIONS

The CET developed from the health navigation program to support health care of men in the pandemic, called “Fala-M@ano-COVID-19”;, involved theoretical and methodological support that allowed the derivation of a viable health navigation program and compatible with the observed reality, based on the target audience’s needs.

It required the deepening of technologies, through the acquisition of technological skills by the executing team (professional navigators) and teamwork with different levels and interdisciplinary/interprofessional areas of activity. Additionally, the technology consisted of: use of nursing taxonomies; interaction; health communication/conversation; digital inclusion in academic education in health; consumption and implementation of scientific evidence; knowledge translation; and technological innovation in nursing and health. “Fala-M@ano-COVID-19”; development proved to be viable, feasible to be operationalized, inclusive, low-cost, driving dialogue and Nursing Process strengthening as a methodological instrument in the production of health care for men.
